# AI Technology: A New Game Changer for the Future Mental Health Industry?

**DOI:** 10.1177/10105395241303790

**Published:** 2024-12-05

**Authors:** Elizabeth Yong, Yen Nee Teo, Kun Hing Yong

**Affiliations:** 1 Indooroopilly State High School, Brisbane, QLD, Australia; 2 Institute of Malaysian and International Studies, The National University of Malaysia, Bangi, Malaysia; 3 School of Medicine and Dentistry, Griffith University, Nathan, QLD, Australia

## Introduction

The global pandemic, coronavirus (COVID-19), had devastating impacts on the world, including the healthcare industry and the general population. Ensuing the onset of the pandemic, symptoms of anxiety and depression experienced a dramatic rise by 25%.^1^ The statistics shown are concerning, as the consequences of failing to address mental health issues can present a largely untreated challenge of suicide, which were found to lead to premature death of approximately 20 years earlier.^2^ At this juncture, the emergence of artificial intelligence (AI) has become a promising tool to be leveraged in the global mental healthcare industry.

## Artificial Intelligence Technology in Mental Healthcare Industry: Benefits

### Enhance Accessibility to and Affordability for Mental Health Support

An alarming majority of individuals with mental health disorders globally fall short in accessing high-quality mental health services due to the scarcity of mental health professionals and affordability. As supported by the latest data of the World Health Organization (WHO),^3^ the ratio of psychiatrists per 100,000 ranges between 0.0 and 48.0 by region. Less than 2 to 48.0 in Europe and Central Asia, 10.5 to 14.7 in North America, less than 10 in Latin America and the Caribbean (excluded 21.7 in Argentina [2016]), 0.3 to less than 10 in East Asia and the Pacific (excluded 11.9 in Japan [2016], 13.5 in Australia [2015], and 28.5 in New Zealand [2016]), 0.2 to 2.0 in Middle East and North Africa, and 0.2 to 0.4 in South Asia.^3^ The utilization of AI applications offers an alternative platform that contributes to ease of access to mental health support, improves flexibility, and is time-saving. Thus, it can be a beneficial source of support for mental health patients, specifically during the COVID-19 pandemic. Increasing the affordability of AI applications is expected to increase the chances of accessibility, specifically for low-income households, thereby becoming one of the solutions to address the gap in supply and demand issues for mental health services. More importantly, those who live in underserved communities or remote areas with a lack of or no access to psychiatrists will be the next group of people who will be benefited. This ensures equal benefits for patients to receive support, therapy care, or psychiatrist-monitored medication, thus reducing the risks of suicide.

### Reduce Stigma and Fear of Judgment

Stigma and discrimination are among the other prominent barriers preventing people from seeking consultation from mental health professionals. The AI applications have the added benefit of being accessible without the need for human interaction. It helps alleviate the fear of judgment, enhance trust in self-disclosure, and increase confidence in sharing mental health-related issues with mental health professionals. Thus, it offers a safe and private space for people seeking support in a judgment-free environment,^4^ which will broaden its accessibility.^5^ Greater gains to the AI applications include promoting self-awareness and providing an alternative pathway to provide support in assessing and diagnosing symptoms that range from mild to moderate on top of digital interventions like telehealth. However, a robust evaluation of the formats of AI application tools is essential to ensure their safety and effectiveness, while minimizing the risks of information misinterpretation. This is crucial given the significance of AI application tools, which are generally designed to support health services including monitoring, diagnosing, and generating personalized treatment plans.^6^

### Improve Clinical Care

The continuous improvement of AI applications in replicating discrete human intelligence skills can significantly benefit mental health practitioners in their clinical care.^7^ In particular, AI applications can improve the accuracy and effectiveness of diagnosis through leveraging the extensive evidence-based data sets to facilitate personalized treatment plans, minimize human errors, and improve decision-making.^8^ However, due to the distinctive diagnostics of mental health illnesses,^9^ capitalizing on a highly specific and sensitive machine learning algorithms is essential to augment the effectiveness of tailored treatment strategies and interventions.

## Artificial Intelligence Technology in Mental Healthcare Industry: Challenges

Integrating AI applications into mental health services has the potential to provide a promising prospect. However, several challenges remain to be addressed before the mental healthcare industry can fully reap the potential benefits of AI technologies. One of the advantages of AI technology is the capability of handling huge data, which can be beneficial for analysis. In this case, data management can be challenging. On one hand, data and knowledge sharing enhance new knowledge and skills among mental health practitioners; on the other hand, it raises concerns regarding ethical issues in relation to patients’ privacy and legal responsibility. Other challenges could be transparency and methodological flaws,^10^ which can significantly affect risks of misinterpretation, outcomes of decision-making, and increase risks of mortality, and hence, trust and integrity of the AI applications in mental health domains. The final challenge is the acceptance level of AI applications by patients, which is dominantly influenced by patients’ confidence toward it as well as the costs of applications of AI technology.

## Conclusion

The COVID-19 epidemic has presented unprecedented challenges and significant changes that have had adverse impacts on the mental health outcomes of people with pre-existing mental health conditions. Despite the development of mental health services, factors including stigma, fear of judgment, accessibility, and affordability contributed to unmet needs for mental health services. The development of AI technology and the prospect of adopting AI technology in mental healthcare as digital medicine are promising to help overcome the current barriers and benefit the mental health industry. Nevertheless, there remain several challenges, including transparency, ethical issues, data management, and risks of misinterpretation, that must be addressed before the mental healthcare industry can fully realize the benefits of AI technologies. Thus, researchers should actively engage in lending their clinical and scientific expertise to help transform mental health practice and enhance care for patients.
